# Prosthetic Cap-Free Implant Restorations: Five-Year Clinical Performance with Mechanical Verification

**DOI:** 10.3390/dj13120586

**Published:** 2025-12-08

**Authors:** Ioan-Achim Borșanu, Laura-Cristina Rusu, Sergiu-Manuel Antonie, Emanuel-Adrian Bratu

**Affiliations:** 1 Clinic of Implant Supported Restorations, “Victor Babes” University of Medicine and Pharmacy, 2 Eftimie Murgu Sq., 300041 Timisoara, Romania; ioan.borsanu@umft.ro (I.-A.B.); ebratu@umft.ro (E.-A.B.); 2 Multidisciplinary Center for Research, Evaluation, Diagnosis and Therapies in Oral Medicine, “Victor Babes” University of Medicine and Pharmacy, 300041 Timisoara, Romania; 3 University Clinic of Oral Pathology, “Victor Babes” University of Medicine and Pharmacy, 300041 Timisoara, Romania

**Keywords:** implant prosthodontics, screw-retained restorations, prosthetic cap, mechanical verification, marginal fit, digital workflow

## Abstract

**Background:** The use of prosthetic caps in screw-retained implant restorations aims to enhance passivity and protect abutment threads; however, these components may increase prosthetic volume and impair esthetics. Advances in high-strength zirconia have raised the question of whether such caps remain necessary. **Methods:** A retrospective clinical analysis was conducted on 20 partial screw-retained zirconia restorations comparing cases fabricated with and without a prosthetic cap. All restorations were followed for 3–5 years. Clinical outcomes included screw stability, marginal adaptation, esthetics (VAS), hygiene access, and biological response. A supplementary mechanical verification was performed on four standardized zirconia crowns fabricated through digital and conventional impression workflows to qualitatively assess their behavior under 30 N·cm torque and compressive loading above 1200 MPa. **Results:** Throughout follow-up, no mechanical or biological complications were recorded in either group. One restoration with a cap required screw re-tightening, while none failed in the cap-free group. Radiographic analysis showed smaller mean marginal gaps in cap-free restorations (0.183 mm) compared to those with caps (0.289 mm; *p* < 0.01). Esthetic satisfaction scores were higher in the cap-free group (VAS = 9.3 ± 0.1 vs. 8.2 ± 0.1; *p* < 0.001). Mechanical verification confirmed that all zirconia crowns tolerated torque and compressive loads without visible fracture or deformation. **Conclusions:** Within the study limitations, cap-free screw-retained zirconia restorations exhibited excellent 5-year clinical stability, improved esthetics, and better hygiene access compared with capped designs. The small-scale mechanical verification supported the clinical findings, indicating that cap omission does not compromise mechanical performance when accurate fit and digital workflow precision are ensured.

## 1. Introduction

Screw-retained implant restorations have become the preferred option in modern implant prosthodontics due to their retrievability, predictable seating, and simplified long-term maintenance compared to cemented alternatives. A systematic review by Wittneben et al. reported that screw-retained prostheses offer improved long-term maintenance and fewer biologic complications, particularly in full-arch and posterior restorations [1]. More recently, studies have confirmed that screw-retained solutions dominate digital workflows, representing 60–70% of implant-supported rehabilitations due to improved precision and retrievability [2,3,4,5].

These advantages include reduced risk of cement-induced peri-implantitis, easier prosthetic retrieval or modification, and decreased risk of subgingival residual cement, a known contributor to implant complications [6,7,8,9,10]. For these reasons, screw-retained designs are now favored in both single-unit and multi-unit cases, particularly when combined with CAD/CAM workflows [11,12,13].

Certain implant systems incorporate intermediary metallic components—often referred to as prosthetic caps—between the abutment and the restoration. These components are intended to enhance passivity, torque stability, and stress distribution across the implant–abutment interface [14,15,16].

However, several clinical and prosthetic limitations are associated with its routine use In esthetic regions, metallic inserts may alter light transmission and compromise the optical properties of translucent ceramics, leading to grayish shadowing and lower esthetic ratings [17,18]. Moreover, the additional vertical and horizontal volume imposed by the cap may complicate soft tissue management, emergence profile design, and interproximal contouring—factors critical for hygiene and long-term peri-implant stability [19]. In limited inter-occlusal space or posterior regions, the prosthetic volume created by the cap may interfere with opposing dentition or require over-contouring, which is both esthetically and functionally undesirable.

From a digital design perspective, the inclusion of additional metallic interfaces reduces design space, increases occlusal clearance requirements, and complicates the scanbody alignment process within CAD/CAM workflows [20,21]. Consequently, clinicians have begun exploring cap-free workflows, particularly with the availability of high-strength monolithic zirconia capable of combining esthetic translucency and flexural strength exceeding 1000 MPa [22,23,24,25].

Despite the growing adoption of metal-free restorative protocols, there is limited clinical evidence on the long-term performance of screw-retained zirconia restorations fabricated without intermediary metallic components. The present study aimed to assess the five-year clinical outcomes of such restorations compared to those fabricated with a prosthetic cap and to verify their mechanical behavior through complementary in vitro testing.

The null hypothesis was that the absence of a prosthetic cap would not significantly influence mechanical performance, marginal adaptation, or clinical success.

## 2. Materials and Methods

### 2.1. Study Design and Ethical Approval

This study employed a mixed-method design combining a retrospective clinical evaluation with a complementary in vitro mechanical verification to analyze the influence of the prosthetic cap on screw-retained zirconia restorations supported by Bredent SKY implants (Bredent GmbH, Senden, Germany). All clinical and laboratory procedures were performed by the same prosthodontic team and laboratory under identical protocols. The sole investigated variable was the presence or absence of the prosthetic cap.

**Figure 1:**
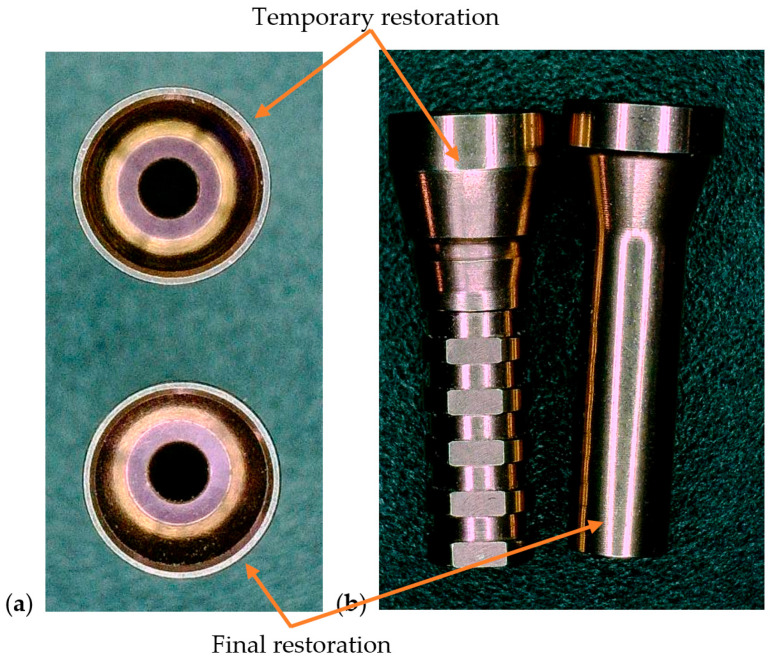
Representative prosthetic caps used as intermediary metallic inserts between the abutment and restoration: (**a**) occlusal view; (**b**) lateral view. The image is illustrative of the component concept; the study’s comparative variable was the presence or absence of such a cap [23].

The clinical study was conducted in accordance with the Declaration of Helsinki and approved by the Ethics Committee for Scientific Research of the “Victor Babeș” University of Medicine and Pharmacy, Timișoara (protocol code 16/21.01.2025). All participants provided written informed consent allowing the scientific use of anonymized clinical and radiographic data. The intermediary prosthetic cap components are shown in Figure 1.

The study was structured in two distinct but complementary parts:

- Clinical Evaluation—A retrospective cohort of 20 screw-retained zirconia restorations placed between 2019 and 2025 was reviewed. Two groups were formed (*n* = 10 each): restorations fabricated with the prosthetic cap and restorations fabricated without the prosthetic cap. All restorations were fabricated from monolithic zirconia and torqued according to manufacturer recommendations. The follow-up period ranged from three to five years.
- Mechanical Verification—A separate set of four in vitro test models was produced to verify the mechanical stability of restorations without caps under controlled conditions. These were not derived from clinical cases.

This dual methodology enabled both real-world clinical assessment and standardized mechanical verification of screw-retained zirconia restorations.

### 2.2. Patient Selection and Clinical Cases

A total of 20 patients rehabilitated with screw-retained partial implant-supported zirconia restorations on SKY implants (Bredent GmbH, Senden, Germany) were retrospectively analyzed from the dental clinic database. All restorations were directly screw-retained to the abutment and were fabricated using a digital CAD/CAM workflow. Treatments were completed between 2019 and 2020, and follow-up continued through early 2025.

Inclusion criteria required patients to be between 35 and 70 years of age, in good general health corresponding to ASA physical status classification I or II, and free of active periodontal disease or any systemic condition that could compromise osseointegration. All patients presented with stable peri-implant soft tissue conditions at the time of prosthetic evaluation. While parafunctional habits such as bruxism were generally considered exclusion criteria, three patients with bruxism under clinical control were included in the study cohort.

Each restoration was supported by at least two implants and designed as a small-span fixed restoration. Although most cases were partial rehabilitations, several extended to full-arch segments following identical clinical and laboratory protocols. In cases where full-arch rehabilitation was performed, the prosthesis was digitally segmented into two or three independent partial restorations to optimize passivity and maintenance. Each segment functioned as a small-span fixed prosthesis, and within each case, at least one restoration was fabricated without the prosthetic cap. No continuous full-arch frameworks were included in the mechanical or statistical evaluation. All restorations were screw-retained on titanium abutments compatible with the Bredent SKY system. All prostheses were fabricated from a high-strength gradient zirconia (IPS e.max ZirCAD Prime, Ivoclar Vivadent, Schaan, Liechtenstein), ensuring uniform material characteristics across the sample.

Based on prosthetic configuration, the cases were divided into two groups:

- Group A: Restorations fabricated with the PKT prosthetic cap (*n* = 10)
- Group B: Restorations fabricated without the PKT prosthetic cap (*n* = 10)

Inclusion and exclusion criteria are summarized in Table 1.

**Figure 2:**
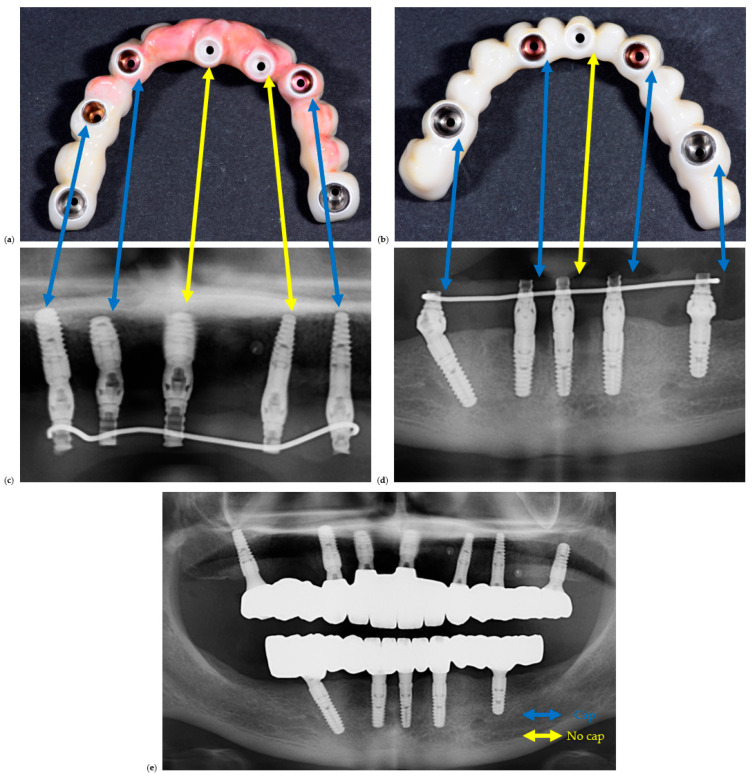
Clinical and radiographic comparison of screw-retained full-arch restorations fabricated with and without prosthetic caps. (**a**) Clinical photo of a maxillary full-arch screw-retained restoration, (**b**) clinical photo of a mandible full-arch screw-retained restoration, (**c**) section of an OPG corresponding to (**a**), (**d**) section of an OPG corresponding to (**b**). Blue lines are between implant sites with caps, while yellow lines are between implant sites with caps. (**e**) is the OPG of the final delivered restorations.

A representative example of restorations fabricated with and without prosthetic caps is illustrated in Figure 2 to demonstrate the restorative configurations compared in this study.

### 2.3. CAD/CAM Design and Fabrication

All restorations were digitally designed in Exocad DentalCad (3.0 and 3.1) software (Exocad GmbH, Darmstadt, Germany) and milled from high-strength gradient zirconia (IPS e.max ZirCAD Prime, Ivoclar Vivadent) using a 5-axis dry-milling unit. Screw-access channels were integrated during design and sealed chairside after insertion. No cementation or luting agents were used at any stage.

Digital and conventional impressions were both used to generate the working models. Intraoral scans were captured with manufacturer-recommended protocols, while conventional impressions employed a medium-body polyether material poured in Type IV dental stone. All models were converted to STL files and processed within the same CAD/CAM software to ensure identical design parameters and eliminate workflow variability.

The design parameters, including connector dimensions, emergence profile, and screw-access alignment, were standardized across all cases to maintain comparability between restorations fabricated with and without the prosthetic cap.

### 2.4. Impression Techniques for Mechanical Verification

To verify the mechanical stability of screw-retained zirconia restorations fabricated without a prosthetic cap, four in vitro models were produced under controlled laboratory conditions. These models simulated posterior edentulous segments and were used solely for mechanical verification, independent of the clinical cohort. Two models were generated via fully digital workflows and two via conventional impression techniques, as described below. All models were fabricated by the same dental laboratory, by the same technician, under the supervision of the prosthodontists, ensuring standardization and reproducibility across all samples.

Digital Workflow

**Figure 3:**
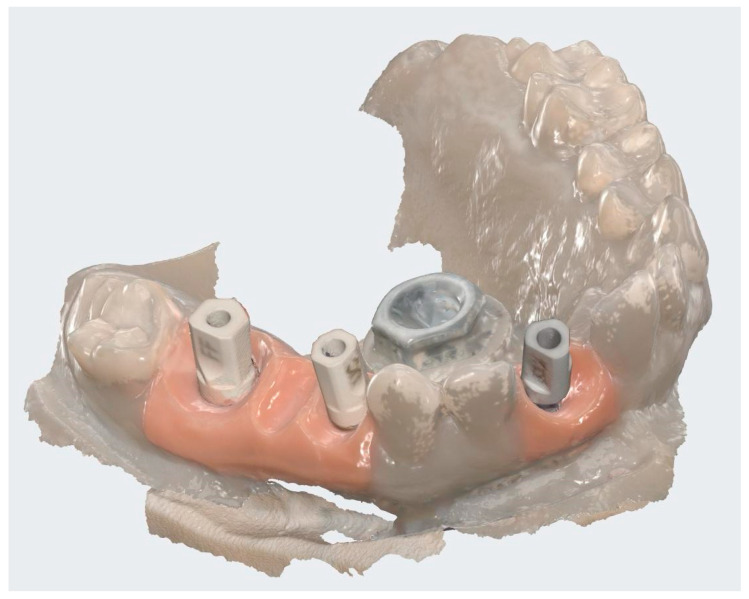
Intraoral scan with Medit.

- Model 1—TRIOS 3 Scanner (3Shape A/S, Copenhagen, Denmark): An intraoral scan was performed using a calibrated digital scanner following the manufacturer’s protocol. Implant scan bodies were positioned and captured to record the exact three-dimensional implant location and angulation.
- Model 2—Medit i700 Scanner (Medit Corp., Seoul, Republic of Korea): A second digital model was obtained using an alternative intraoral scanner with identical scanning procedures and scan-body positioning to ensure comparability (Figure 3).

Conventional Workflow

**Figure 4:**
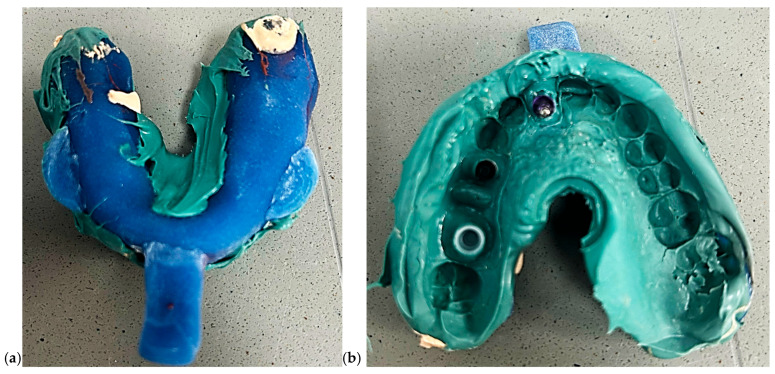
Pictures of closed tray impression. (**a**) External view of the closed-tray impression showing the impression material and tray configuration. (**b**) Internal view of the same closed-tray impression demonstrating the captured implant transfer copings and surrounding anatomical details.

**Figure 5:**
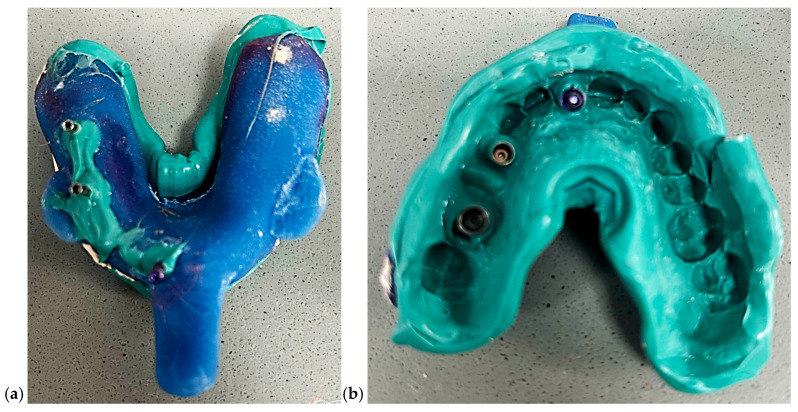
Pictures of open tray impression. (**a**) External view of the open-tray impression showing the impression material, tray perforations, and visible screw access channels. (**b**) Internal view demonstrating the captured open-tray impression copings and detailed reproduction of the surrounding anatomical structures.

- Model 3—Closed Tray Technique: A closed tray impression was taken using medium-bodied polyether material (Impregum™ Penta, 3M ESPE, Neuss, Germany) (Figure 4). The impression coping was unscrewed post-set, and a laboratory analog was inserted into the impression before pouring. This method is often favored for its simplicity in single-unit posterior cases.

- Model 4—Open Tray Technique with Splinted Copings: An open tray impression was taken using splinted impression copings joined with light-cured composite resin (e.g., GC Pattern Resin LS) and reinforced with flowable resin to minimize coping movement (Figure 5). The same polyether material was used to ensure consistent dimensional stability.

CAD/CAM Fabrication

**Figure 6:**
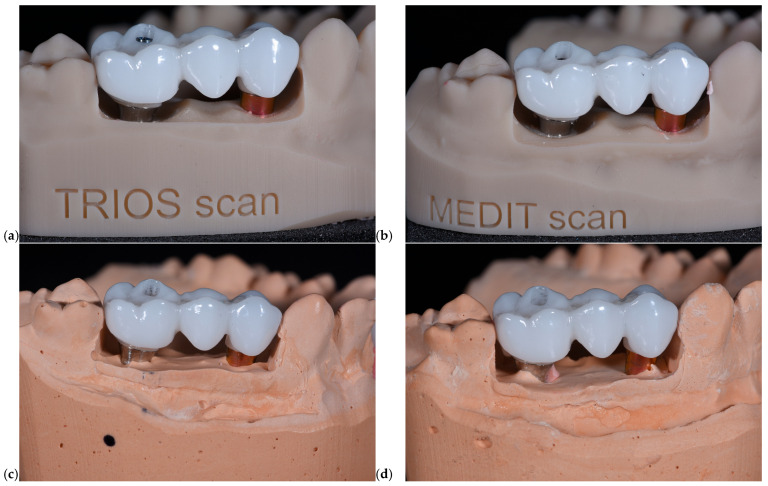
Zirconia restorations for the model scanned with TRIOS scanner (**a**), Medit scanner (**b**), and for models performed after closed tray (**c**) and open tray (**d**) impressions.

All models, digital and conventional, were converted or scanned into STL format and imported into Exocad software platform to eliminate variability between workflows. A standardized anatomic design was applied across all samples, and restorations were designed as monolithic zirconia crowns with integrated screw channels. No manual modifications were made to maintain consistency. In Figure 6 photos of each type of restoration is presented.

**Figure 7:**
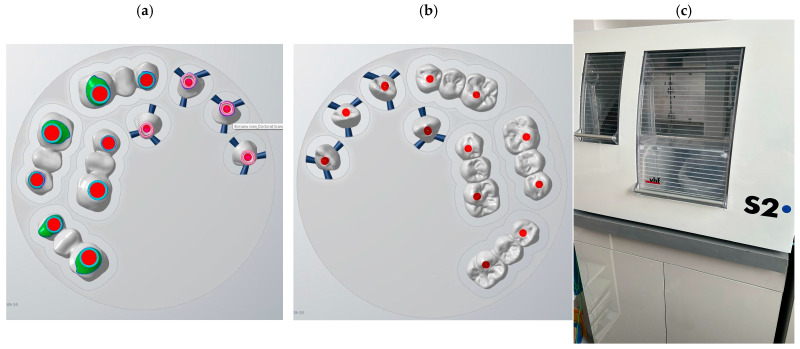
Print screens from milling software with restorations ready for milling (**a**,**b**), and the milling machine vhf S2 (**c**).

Restorations were designed as monolithic zirconia crowns with direct screw access, without the use of the PKT cap, and milled using a 5-axis system. The same design was applied across all models to maintain consistency. The digital design is arranged on zirconia blocks from milling software and then sent to the vhf S2 milling machine (vhf camfacture AG, Ammerbuch, Germany) (Figure 7).

These restorations were subsequently subjected to the mechanical verification protocols described in Section 2.5, which evaluated their resistance to excessive torque (≥30 N·cm) and to compressive loads exceeding typical masticatory forces.

**Figure 8:**
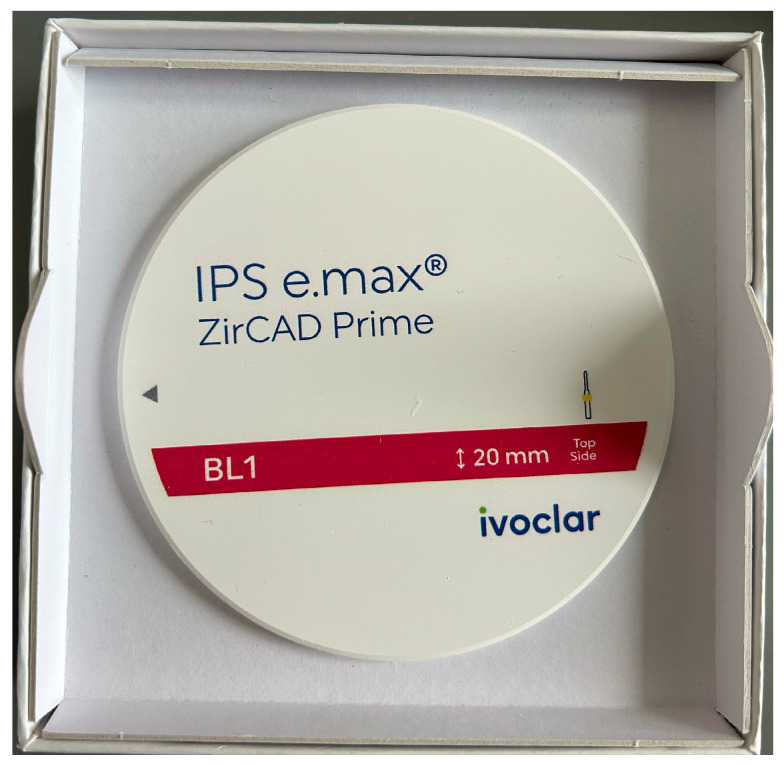
IPS e.max ZirCAD Prime utilizes a continuous gradient structure created through Gradient Technology (GT), seamlessly transitioning from a highly translucent incisal zone to a high-strength dentin core.

The purpose of this experimental phase was to verify the structural reliability of restorations fabricated without prosthetic caps, produced through different impression workflows and tested under extreme mechanical stress conditions. All test restorations were milled from high-strength gradient zirconia (IPS e.max ZirCAD Prime, Ivoclar Vivadent) (Figure 8), ensuring identical material characteristics across all models. The material exhibits a graded microstructure with flexural strength values reaching approximately 1200 MPa in the dentin zone and increasing translucency toward the incisal region, providing an appropriate balance of mechanical performance and esthetics [24,25,26].

All restorations used for mechanical testing in this study were designed to ensure that occlusal load distribution occurred primarily over the high-strength core region, maximizing resistance under exaggerated compressive forces. In the following table (Table 2), there it is a technical comparison between the impression models.

### 2.5. Mechanical Verification

Mechanical testing was conducted to evaluate the resistance and structural integrity of screw-retained partial zirconia restorations fabricated without the PKT prosthetic cap when subjected to mechanical stress conditions exceeding typical intraoral loads.

These tests were designed to simulate clinical extremes, including overtightening torque and masticatory overload, to verify the mechanical safety margin and long-term viability of using uncapped restorations in demanding clinical situations. Two independent test series were performed: torque resistance and load-to-failure assessment. All specimens were manufactured under identical CAD/CAM conditions and secured to titanium abutments compatible with the same implant system. Each restoration was supported by at least two implants to ensure comparable load distribution.

#### 2.5.1. Torque Resistance Test

**Figure 9:**
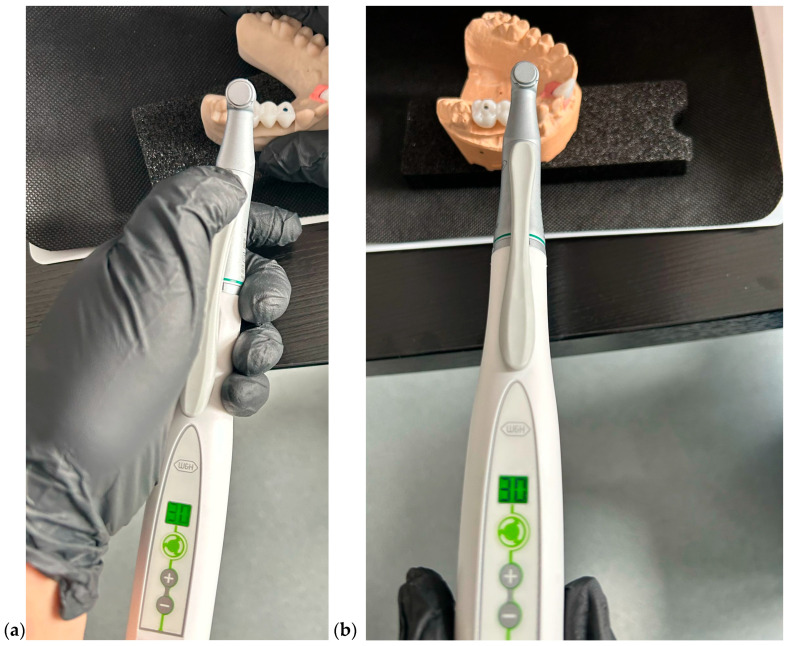
Torque resistance evaluation at 30 N·cm for (**a**) molar and (**b**) premolar partial restoration.

Each restoration was screw-retained onto its corresponding model and subjected to a torque of 30 N·cm (Figure 9), which exceeds the standard clinical recommendation of 20 N·cm for SKY implants (Bredent GmbH). The purpose of this test was to simulate a worst-case clinical scenario in which over-torque may occur during seating or maintenance.

The torque application was performed manually using a calibrated digital torque wrench. During and after torquing, restorations were evaluated for:

- Marginal integrity
- Surface cracks or deformation
- Abutment thread distortion or damage

The selection of 30 N·cm was based on the manufacturer’s maximum allowable torque for titanium abutments, representing an intentionally elevated condition to assess the structural stability of the zirconia-abutment interface.

#### 2.5.2. Load to Failure Test

**Figure 10:**
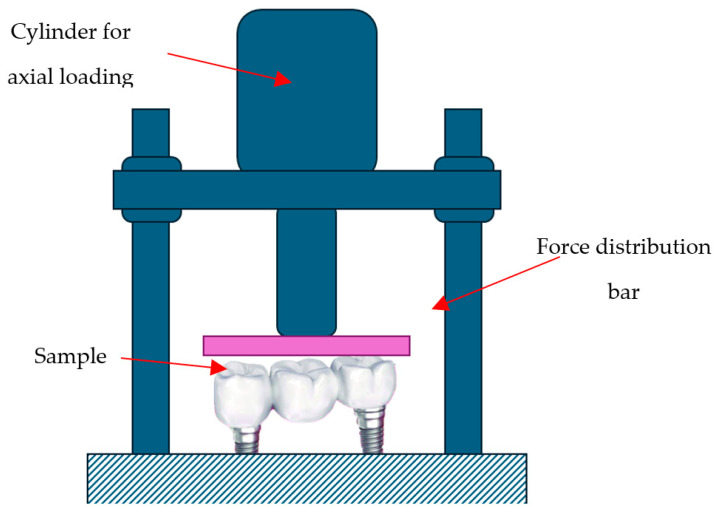
Static compressive testing setup for mechanical verification.

Following the torque resistance protocol, the same zirconia restorations were subjected to static compressive loading (Figure 10) using a testing machine at the Department of Mechanical Engineering, Politehnica University of Timișoara. This procedure aimed to determine the ultimate load capacity of restorations fabricated without the PKT prosthetic cap, under stress conditions intentionally exceeding normal functional limits.

Test parameters included:

- Force direction: distributed with the help of a metallic bar as can be seen in the schematics
- Environment: Dry, room temperature, standard laboratory conditions

Outcome measures:

- Maximum compressive load (N)—120–150 N
- Post-load inspection protocol including visual, microscopic, and radiographic assessment of marginal areas

Static compressive loading was applied using a metallic distribution bar positioned across the occlusal surfaces to ensure even force transmission to both abutments. The universal testing machine recorded the maximum load (N) at failure. Because the load was distributed along the entire restoration, stresses in MPa were not directly computed; results are therefore reported as maximum load values (N), which directly indicate mechanical resistance under extreme conditions. Assuming an estimated contact area of approximately 10 mm^2^ per abutment, the equivalent compressive stress would correspond to about 120 MPa, well below the material’s flexural strength of 1200 MPa specified in the manufacturer’s datasheet for IPS e.max ZirCAD Prime. The applied compressive loads (approximately 1250–1300 N) were intentionally higher than physiological and parafunctional bite forces (typically 70–700 N) to establish a safety margin and validate structural stability under exaggerated loading.

Assuming an approximate contact area of 10 mm^2^ per abutment, the equivalent compressive stress would be around 120 MPa per abutment. However, since the load was applied through a distribution bar rather than a localized contact, this calculated stress value is only an approximation and should be interpreted qualitatively.

The applied compressive forces (≈1250–1300 N) intentionally exceeded the normal range of physiological and parafunctional bite forces (70–700 N) to introduce a safety margin and verify the restorations’ structural reliability relative to the material’s specified flexural strength (up to 1200 MPa, as reported by the manufacturer for IPS e.max ZirCAD Prime).

### 2.6. Clinical Evaluation

A total of 20 patients received screw-retained partial zirconia restorations on SKY implants (Bredent GmbH) since 2019, with clinical follow-up extending through 2025. All restorations were fabricated from IPS e.max ZirCAD Prime (Ivoclar Vivadent), and at least one implant in each patient from Group B (without PKT cap) was restored without an intermediary Ti-base.

Clinical assessments were conducted at baseline, 6 months, and annually thereafter by the same prosthodontic team. The analysis included both qualitative and quantitative parameters, with standardized photographic and radiographic documentation. Each periapical radiograph was first calibrated using a known reference length—typically the diameter of the implant platform (e.g., 4.0 mm), which was confirmed based on manufacturer specifications. A straight line was drawn across the known length on the image using the Line Tool. The Set Scale function was then used to input the real-world distance and units (mm). The scale was adjusted for each image in order to apply consistent calibration across all images.

Once calibrated, the marginal gaps between the abutment and the prosthetic interface were measured at multiple points on each radiograph. Using the Zoom Tool, the crown–implant interface was enlarged to ensure accurate selection of measurement points. The Line Tool was used to measure the perpendicular distance between the zirconia crown margin and the implant platform at visible gap locations. Each restoration was measured. The Measure function was applied, and data was recorded in the Results Table.

Esthetic evaluation using clinician and patient VAS scores (0–10 scale) was performed during recall visits, along with evaluation of prosthetic integrity, marginal adaptation, and hygiene accessibility. Radiographs obtained during routine check-ups were used for marginal evaluation, and all measurements were standardized using ImageJ Fiji software (National Institutes of Health, Bethesda, MD, USA).

### 2.7. Statistical Analysis

All statistical analyses were performed to compare the clinical and radiographic outcomes between restorations fabricated with and without the prosthetic cap. The primary outcome variable was the marginal gap (mm), measured on standardized and calibrated radiographs using ImageJ software (National Institutes of Health, Bethesda, MD, USA). Secondary outcomes included the esthetic satisfaction score (Visual Analog Scale, 0–10), recorded as the mean value between clinician and patient assessments, and categorical clinical parameters such as screw re-tightening, biological complications, and restoration reprocessing events.

For each restoration, multiple radiographic measurements were taken at the abutment–crown interface and averaged per case. The resulting data were organized in Microsoft Excel (Microsoft Corp., Redmond, WA, USA) and analyzed using the StatsKingdom online platform (https://www.statskingdom.com, accessed on 20 April 2025). All test outcomes were double-checked in JASP (version 0.95, University of Amsterdam, Amsterdam, The Netherlands) to verify statistical concordance.

Continuous data were expressed as mean ± standard deviation (SD) with 95% confidence intervals (CI). The Shapiro–Wilk test was applied to assess the normality of data distribution. When normality assumptions were met, comparisons between groups were conducted using the independent samples *t*-test; otherwise, the Mann–Whitney U test was applied. To confirm the stability of results, additional one-way ANOVA and Kruskal–Wallis H tests were performed. No correction for multiple testing was necessary, as the marginal gap represented the predefined primary endpoint, and the other analyses were exploratory.

A two-tailed significance level of α = 0.05 was used for all comparisons. This combined parametric and non-parametric approach was chosen to ensure statistical robustness and to minimize assumption bias in a small, balanced clinical sample. The use of both StatsKingdom and JASP platforms ensured methodological transparency and verification of analytical accuracy.

## 3. Results

### 3.1. Mechanical Testing Results

Mechanical testing was performed on four categories of zirconia restorations fabricated without the PKT prosthetic cap, each corresponding to a different impression technique. All restorations were subjected to both over-torque and compressive stress testing, as described in Section 2.4.

Torque Resistance

All crowns tolerated a torque of 30 N·cm (exceeding the standard 20 N·cm) without fracture, screw loosening, or marginal deformation. No cracking, chipping, or misfit was observed after over-torqueing.

**Figure 11:**
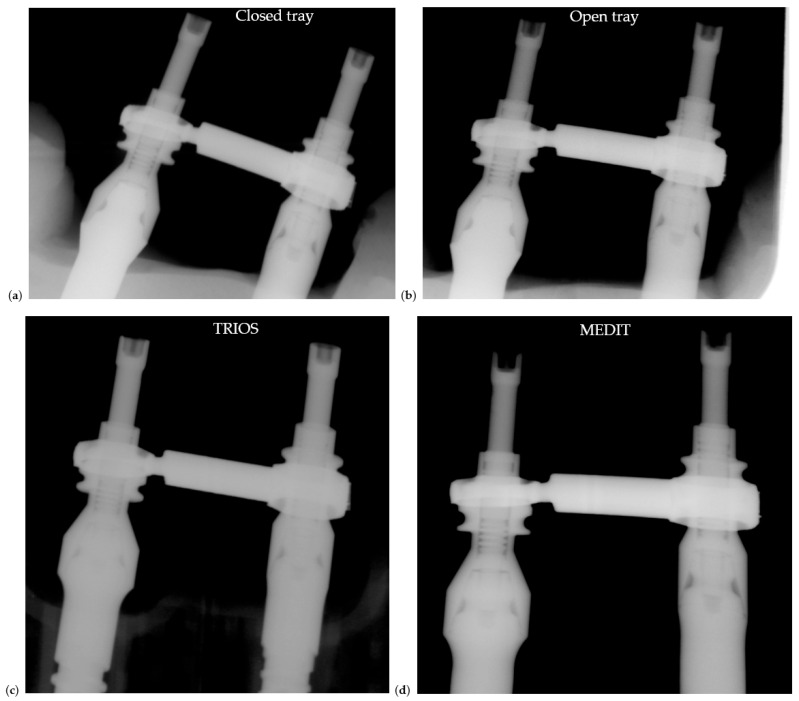
Radiographic comparison of soldered test bars on models fabricated with different impression techniques: (**a**) closed tray conventional impression, (**b**) open tray impression, (**c**) TRIOS scan and (**d**) MEDIT scan.

All models were fitted with the same soldered verification bar (Figure 11). These radiographs were used to assess passive fit and impression accuracy under identical test conditions.

**Figure 12:**
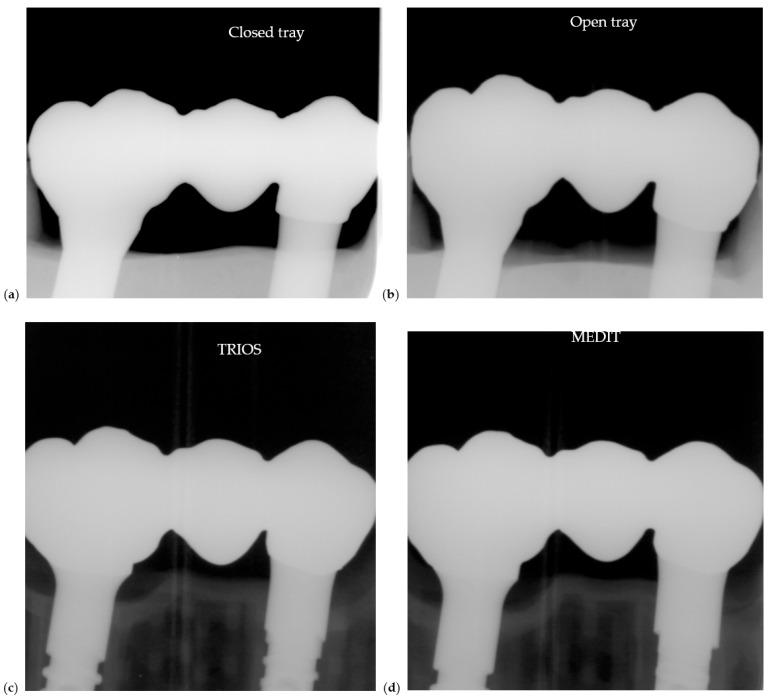
Radiographic evaluation of Zirconia restorations seated on models fabricated with different impression techniques: (**a**) closed tray conventional impression, (**b**) open tray impression, (**c**) TRIOS scan and (**d**) MEDIT scan.

Radiographs were taken to assess passive fit, abutment–restoration interface alignment, and potential marginal gaps under clinical torque values (Figure 12).

Load-to-Failure Testing

Under compressive force application using a universal testing machine, all test crowns withstood forces exceeding 1200 N without exhibiting catastrophic structural failure.

**Figure 13:**
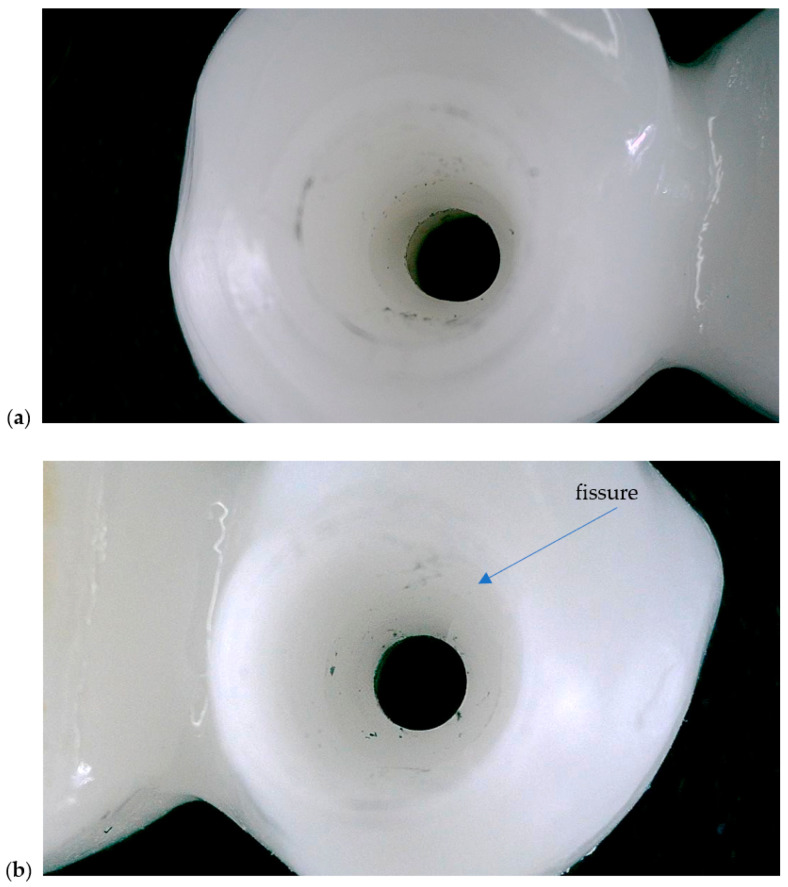
Pictures of the internal surface of Zirconia test models after mechanical loading. (**a**)—open tray model, (**b**)—MEDIT model.

No visible fractures or bulk material failure occurred in any of the test models during or after load application. However, a superficial fissure was observed in the screw access channel of the crown fabricated from the MEDIT i700 digital impression (Medit Corp., Seoul, Republic of Korea), suggesting a potential area of localized stress concentration. The fissure did not propagate or compromise the overall integrity of the crown but was detectable under microscopic inspection (Figure 13).

Only minimal post-load polishing marks and surface wear were observed across the other samples, with all restorations maintaining structural stability throughout the mechanical testing protocol.

In both images, toolpath and torque application marks are visible along the inner walls of the screw channel. The summary of mechanical testing on test models are exposed in Table 3.

### 3.2. Clinical Outcomes

Clinical evaluation was performed on 20 restorations in total, with 10 cases in each group (with and without the PKT cap). All restorations were followed for 3 to 5 years post-insertion.

The Group B restorations (without the PKT cap) demonstrated equal or superior outcomes across several parameters, including hygiene accessibility, esthetics, and post-delivery adjustability. No biological complications were observed in either group. Clinical outcomes over 3–5 years of follow-up is summarized in Table 4.

**Figure 14:**
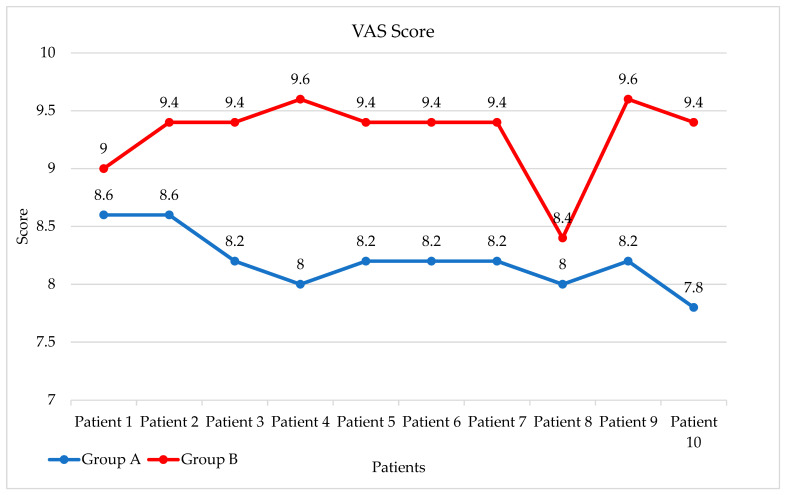
Mean VAS Score for each patient from each group.

As illustrated in Figure 14, the VAS results showed a consistent difference between the two groups. Group B (restorations without the PKT cap) achieved significantly higher mean scores (mean = 9.3 ± 0.13) compared to Group A (mean = 8.2 ± 0.10), particularly in esthetically sensitive regions.

**Figure 15:**
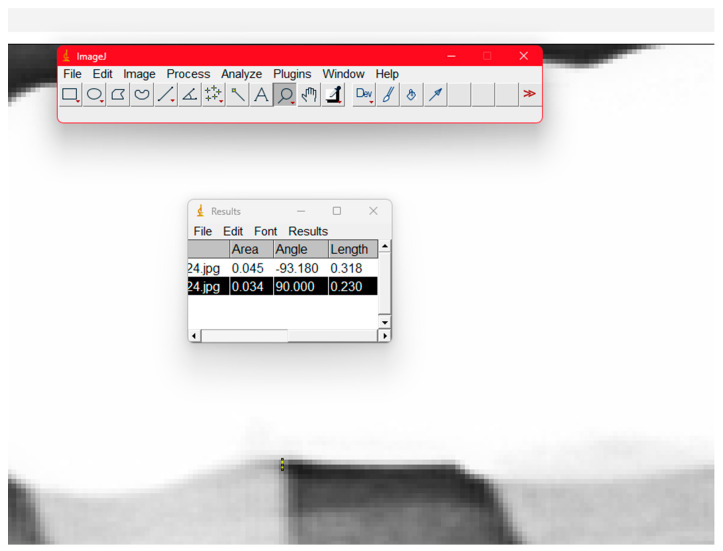
Measurement of a marginal gap on a section from a panoramic radiography with ImageJ software.

In order to illustrate the limitations of radiographic marginal-gap assessment, Figure 15 shows an example of how ImageJ software was used to obtain semi-quantitative measurements from a panoramic radiograph. The measurement tool identifies a visible interface between the abutment and the restoration, and linear calibration allows estimation of the apparent gap width.

To evaluate marginal adaptation, radiographs were analyzed using ImageJ software (National Institutes of Health, USA), a validated open-source tool for quantitative image analysis.

An independent samples *t*-test confirmed the statistical significance of this difference (t = 19.16, *p* < 0.0001). A non-parametric Mann–Whitney U test was also conducted, yielding consistent results (U = 0.0, *p* < 0.0001), thus reinforcing the robustness of the findings regardless of distributional assumptions. These results underscore the esthetic and functional superiority of zirconia restorations fabricated without the PKT cap from the patient’s perspective. Table 5 is showing the measurements for marginal gap differences for each group.

To assess the accuracy of marginal adaptation, ImageJ software was used to measure radiographic gaps in restorations torqued to 30 N·cm. Quantitative analysis revealed a mean marginal gap of 0.289 mm in Group A and 0.183 mm in Group B, suggesting improved fit in the restorations fabricated without the prosthetic cap.

To statistically validate these observations, a comprehensive set of parametric and non-parametric tests was performed:

1. Independent Samples *T*-Test
  - t = 4.3174, *p* = 0.0009
  - Restorations in Group B exhibited significantly smaller marginal gaps, confirming visual and radiographic observations of improved adaptation.
2. Mann–Whitney U Test
  - U = 90.0, *p* = 0.0028
  - The non-parametric test upheld the significance of the difference, confirming the trend in marginal fit even with small sample sizes and without assuming normality.
3. One-Way ANOVA
  - F = 20.06, *p* = 0.0004
  - ANOVA analysis reinforced the influence of prosthetic configuration on marginal gap magnitude, supporting the benefit of cap-free design.
4. Kruskal–Wallis H Test
  - H = 9.9939, *p* = 0.0025
  - A statistically significant difference in rank-based marginal adaptation further supports the finding that restorations without the PKT cap achieve better clinical adaptation.

**Figure 16:**
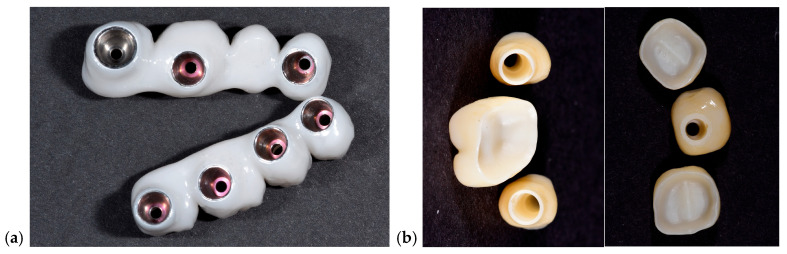

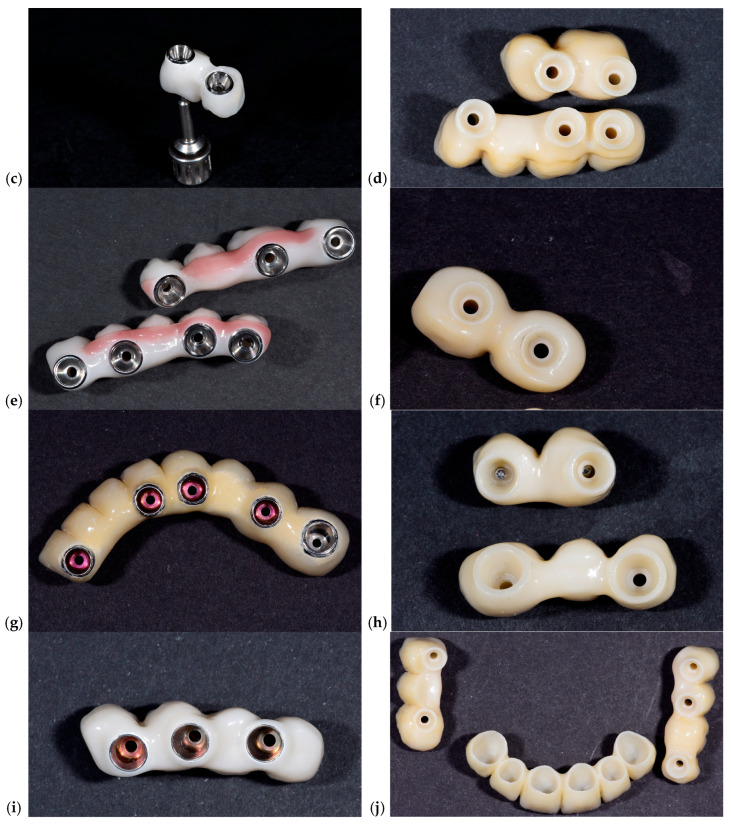
Pictures of dental restorations with (**a**,**c**,**e**,**g**,**i**) or without (**b**,**d**,**f**,**h**,**j**) the PKT from cases selected for this study.

**Figure 17:**
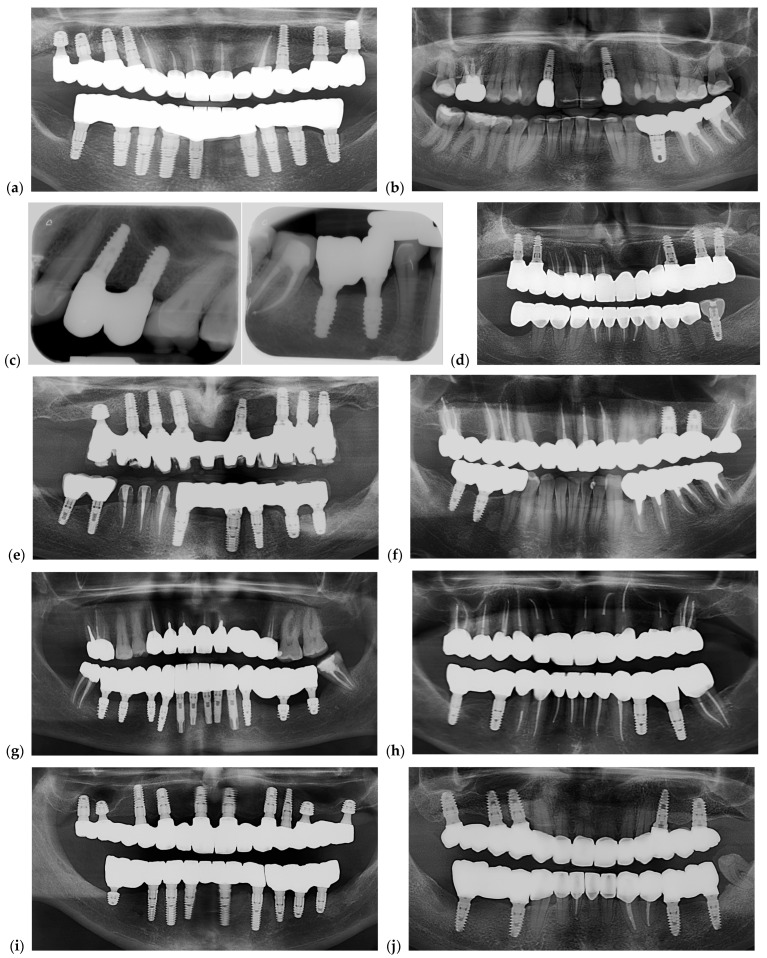
Representative radiographs illustrating the seating and adaptation of the analyzed restorations after fixation. (**a**,**c**,**e**,**g**,**i**) Restorations with the PKT cap; (**b**,**d**,**f**,**h**,**j**) restorations without the PKT cap. Panoramic radiographs were primarily used to document overall fit and implant distribution, while periapical radiographs (panel (**c**)) were included only in one representative case to confirm marginal seating and screw engagement at higher resolution. All radiographs served as qualitative confirmation of restoration fit following final torque, not for quantitative gap measurement. Each radiograph corresponds to photos in Figure 16.

Photos of 5 restorations from selected cases for each group are presented in Figure 16. Radiographic images are shown in Figure 17. Each image from Figure 16 is corresponding with the same position of the Figure 17.

Both panoramic and periapical radiographs were used to verify restoration seating after fixation. Panoramic views documented overall prosthetic alignment, while periapical images (in one case) confirmed marginal fit and screw engagement at higher resolution

No biological complications (e.g., peri-implantitis, bleeding on probing) were observed in either group, and marginal bone levels remained stable on radiographs throughout the observation period.

The following clinical outcome parameters were assessed:

- Prosthetic Stability: No incidents of screw fracture or loss of retention were reported in either group. One restoration in Group A (with cap) required screw re-tightening.
- Marginal Adaptation: All restorations demonstrated clinically acceptable marginal fit with no detectable radiographic gaps or microleakage.
- Esthetic Evaluation: Mean VAS scores were higher in Group B (without cap), particularly in anterior regions.
- Hygiene Accessibility: Patients in Group B reported easier hygiene maintenance and reduced plaque accumulation.
- Biological Parameters: No peri-implantitis, soft tissue inflammation, or mucosal recession was observed. Bleeding on probing (BOP) remained minimal in both groups.
- Adjustability: Two Group B restorations were reglazed after minor wear or contact adjustment; this was not feasible in Group A due to the metallic cap.

Overall, restorations fabricated without the PKT cap achieved comparable mechanical and biological outcomes to those fabricated with the cap, with higher esthetic and hygiene-related scores.

## 4. Discussion

This study aimed to evaluate the clinical and mechanical performance of screw-retained partial zirconia restorations fabricated without the use of the PKT prosthetic cap, a component routinely recommended in the SKY implant system (Bredent GmbH). Using long-term clinical follow-up and complementary mechanical testing, results showed that omitting the PKT cap did not affect prosthetic stability, marginal fit, or biological response. At the same time, cap-free restorations demonstrated advantages in esthetics, hygiene access, and adjustability after insertion.

No fractures were recorded even under compressive forces exceeding 1200 MPa, surpassing the stated flexural strength of the material (ranging from 750 MPa in the incisal third to 1100 MPa near the prosthetic interface, per manufacturer data). This confirms that cap-free zirconia restorations can endure extreme loading conditions without structural failure.

### 4.1. Mechanical Reliability Without the PKT Cap

Mechanical testing indicated that monolithic zirconia restorations fabricated without the PKT cap resisted torque values and compressive loads above typical masticatory thresholds. The gradient microstructure of IPS e.max ZirCAD Prime enables both strength and translucency within a single block, ensuring high load resistance without visible deformation or screw loosening at 30 N·cm torque. These findings indicate that the PKT cap may not be required to achieve mechanical stability when restorations are digitally designed and manufactured under controlled conditions.

However, it is important to note that one of the crowns fabricated on the MEDIT-derived model exhibited a fine fissure at the internal screw channel after torque and load application. The localized crack may reflect stress concentration related to slight seating inaccuracies from scanning. This emphasizes the importance of accurate digital impressions to avoid internal tension in cap-free designs.

Previous studies have highlighted the relevance of stress distribution at the abutment–restoration interface in preventing mechanical complications [27,28]. These observations are consistent with reports on full-contour zirconia frameworks [29,30] and on the long-term stability of advanced dental ceramics [31].

### 4.2. Clinical Performance and Hygiene Benefits

The five-year follow-up confirmed satisfactory outcomes for both groups, with cap-free restorations showing improved esthetics and easier hygiene maintenance. Eliminating the metallic cap reduced light reflection and allowed more anatomical emergence contours, consistent with prior observations on monolithic zirconia in esthetic areas [32,33].

Patients also reported improved hygiene access and easier cleaning due to the reduced bulk of the restoration. This may contribute positively to long-term soft-tissue stability, as previously noted in peri-implant health studies [34].

### 4.3. Post-Insertion Adjustability

Two cap-free restorations were reglazed or adjusted after delivery, demonstrating feasible reprocessing without damage. This thermal compatibility advantage is absent in restorations containing metallic components such as the PKT cap. Such flexibility may be useful in managing occlusal wear or contact loss over time.

### 4.4. Clinical Relevance in Digital Workflows

The findings of this study have significant implications for modern digital prosthodontics. As CAD/CAM workflows become standard in implant dentistry, the precision of milled restorations and high-performance multilayered zirconia reduces the necessity for additional metallic substructures. The omission of prosthetic caps simplifies both design and manufacturing, while allowing individualized emergence profiles and enhanced esthetic control, consistent with the ongoing shift toward metal-free, digitally optimized restorations [35,36]. However, the presence of a micro-fissure in the MEDIT-derived model underscores the critical importance of scanning accuracy. While TRIOS-based restorations demonstrated optimal fit both radiographically and clinically, minor discrepancies in MEDIT and conventional workflows may lead to localized stress accumulation. A possible handling inaccuracy or scan data limitation cannot be excluded. Therefore, when precision in digital impressions cannot be assured, maintaining the prosthetic cap may remain a prudent approach.

### 4.5. Limitations and Future Directions

The study is limited by its small cohort and the absence of randomized group allocation. Mechanical testing did not include cyclic fatigue or thermocycling, limiting extrapolation to long-term intraoral performance. Further multicenter studies with larger sample sizes and dynamic loading protocols are warranted. In Table 6 are presented the advantages and disadvantages of omitting the PKT prosthetic cap.

## 5. Conclusions

Within the limitations of this study, screw-retained partial zirconia restorations fabricated without the PKT prosthetic cap exhibited high mechanical strength and favorable clinical outcomes over a five-year observation period. The restorations withstood torque and compressive forces exceeding the reported flexural strength of the material, without evidence of structural compromise, marginal discrepancy, or screw instability.

Clinically, omission of the PKT cap was associated with distinct esthetic and functional benefits. In anterior regions, the absence of a metallic interface enhanced light transmission and eliminated grayish opacity, improving the optical integration of monolithic zirconia restorations. The reduced prosthetic volume and simplified emergence profiles facilitated better hygiene accessibility, with patients reporting easier cleaning in interproximal and submucosal areas—factors that may contribute to peri-implant tissue stability.

From a mechanical standpoint, the isolated occurrence of a micro-fissure in one MEDIT-derived test model highlights the importance of precise fit and accurate digital impression techniques, particularly in workflows that omit passive intermediary components. These findings suggest that the PKT prosthetic cap may not be universally required when high-strength zirconia and validated digital workflows are employed. Nonetheless, meticulous planning, scanner calibration, and case-specific evaluation remain essential to ensure long-term success.

Future studies involving larger, randomized patient cohorts and incorporating cyclic fatigue and thermomechanical testing are warranted to further validate the biomechanical and biological performance of prosthetic cap-free zirconia restorations. Such evidence would support the broader clinical adoption of simplified, metal-free implant prosthodontic protocols.

## Figures and Tables

**Table 1:** Summary of Patient Selection Criteria.

Category	Criteria
Study Population	20 patients treated with screw-retained partial zirconia restorations on Bredent SKY implants
Follow-up Duration	Minimum of 3 years, some cases reached 5 years
Inclusion Criteria	Good general health (ASA I–II), no active periodontal disease, stable implants, direct screw retention
Exclusion Criteria	Uncontrolled systemic diseases, untreated bruxism, implant mobility, cement-retained restorations

**Table 2:** Technical Comparison of Impression Models.

Model Type	Mean Procedure Time (min)	Relative Cost	Workflow Type	Estimated Dimensional Accuracy (µm)
TRIOS 3Shape (Digital)	5–10	High (equipment investment)	Fully digital intraoral scan	20–30 µm
Medit i700 (Digital)	5–10	Moderate	Fully digital intraoral scan	30–40 µm
Closed Tray (Conventional)	10–15	Low	Polyether impression/Type IV stone model	50–70 µm
Open Tray with Splinted Copings (Conventional)	15–20	Moderate–High	Splinted impression/Type IV stone model	30–40 µm

**Table 3:** Summary of Mechanical Testing on Test Models (Without PKT Cap).

Test Type	Objective	Test Conditions	Outcome
Torque Resistance	Assess resistance to over-torque beyond clinical norms	Manual torque of 30 N·cm (vs. recommended 20 N·cm)	No deformation, fracture, or screw loosening observed
Compressive Load-to-Failure	Simulate excessive occlusal/masticatory force	Universal testing machine; vertical and angled load; force > 1200 N	No structural failure; zirconia tolerated stress beyond the declared 1200 MPa, except for the MEDIT restoration, where a small crack was observed under microscopic inspection.
Material Specification	Reference flexural strength of zirconia disk layers	IPS e.max ZirCAD Prime: up to 1200 MPa in functional zone	Test crowns exceeded the manufacturer-stated strength limit without fracture

**Table 4:** Summary of Clinical Outcomes Over 3–5 Years of Follow-Up.

Parameter	Evaluation Method	Group A (With Cap)	Group B (Without Cap)
Screw Stability	Manual checking, torque feedback	1 case required re-tightening	No incidents
Marginal Fit	Radiographs, explorer probing	Clinically acceptable	Clinically acceptable
Esthetic Integration	VAS score (0–10 scale)	Avg. VAS 8.2	Avg. VAS 9.3 (notably better in anterior)
Hygiene Accessibility	Patient feedback, soft tissue inspection	Acceptable, sometimes compromised	Improved access, easier hygiene
Biological Complications	BOP, peri-implant probing	None	None
Adjustability	Ability to reglaze or adjust contact points	Limited (metal cap interfered)	Reprocessed in 2 cases with success

**Table 5:** Marginal Gap differences for each group.

Mean Value for Group A	Length (mm)	Mean Value for Group B	Length (mm)
a	0.340	b	0.234
c	0.413	d	0.146
e	0.159	f	0.178
g	0.300	h	0.178
i	0.217	j	0.193
k	0.280	l	0.185
m	0.320	n	0.178
o	0.248	p	0.139
q	0.286	r	0.160
s	0.330	t	0.240
Mean	0.289	Mean	0.183

**Table 6:** Advantages and Disadvantages of Omitting the PKT Prosthetic Cap.

Aspect	Advantages (Without PKT Cap)	Disadvantages/Considerations
Esthetics	Improved translucency; no metallic shadowing in the anterior zone	Requires precise design to ensure sufficient support
Hygiene Access	Easier cleaning. Reduced bulk improves interproximal access	Risk of over-contouring if emergence profile not optimized
Mechanical Resistance	High load resistance (>1200 MPa) confirmed; no structural failure under testing	Dependent on adequate wall thickness and material integrity
Adjustability/Repair	Can be reglazed or adjusted after delivery	Inapplicable when metallic cap is used
Digital Workflow	Simplifies CAD design; fewer steps	Deviates from manufacturer recommendations
Passivity/Fit	Maintained through precise CAD/CAM milling	Lacks intermediary buffer effect of PKT cap

## Data Availability

All anonymized clinical and radiographic materials relevant to the study are included within the manuscript. Any additional underlying information pertains to patient medical records and contains personally identifiable health data protected under the General Data Protection Regulation (GDPR; Regulation (EU) 2016/679). For this reason, raw datasets cannot be made publicly available. However, the authors remain open to clarifying any methodological details or answering specific questions related to data interpretation or study replication, within the limits permitted by ethical approval and data-protection regulations.

## Abbreviations

The following abbreviations are used in this manuscript:

PKT	Polyetheretherketone titanium
CAD/CAM	Computer-Aided Design/Computer-Aided Manufacturing
ASA	American Society of Anesthesiologists
STL	Stereolitography
GT	Gradient Technology
VAS	Visual Analogue Scale
BOP	Bleeding on probing
